# Structured Populations of *Sulfolobus acidocaldarius* with Susceptibility to Mobile Genetic Elements

**DOI:** 10.1093/gbe/evx104

**Published:** 2017-06-14

**Authors:** Rika E. Anderson, Angela Kouris, Christopher H. Seward, Kate M. Campbell, Rachel J. Whitaker

**Affiliations:** 1 Carl R. Woese Institute for Genomic Biology, University of Illinois at Urbana-Champaign; 2 Biology Department, Carleton College, Northfield, Minnesota; 3 Energy, Bioengineering and Geomicrobiology Group, University of Calgary, Alberta, Canada; 4 U.S. Geological Survey National Research Program, Boulder, Colorado; 5 Department of Microbiology, University of Illinois at Urbana-Champaign

**Keywords:** *Sulfolobus* species, pangenome, population dynamics, genome divergence, geothermal habitat, immigration

## Abstract

The impact of a structured environment on genome evolution can be determined through comparative population genomics of species that live in the same habitat. Recent work comparing three genome sequences of *Sulfolobus acidocaldarius* suggested that highly structured, extreme, hot spring environments do not limit dispersal of this thermoacidophile, in contrast to other co-occurring *Sulfolobus* species. Instead, a high level of conservation among these three *S. acidocaldarius* genomes was hypothesized to result from rapid, global-scale dispersal promoted by low susceptibility to viruses that sets *S. acidocaldarius* apart from its sister *Sulfolobus* species. To test this hypothesis, we conducted a comparative analysis of 47 genomes of *S. acidocaldarius* from spatial and temporal sampling of two hot springs in Yellowstone National Park. While we confirm the low diversity in the core genome, we observe differentiation among *S. acidocaldarius* populations, likely resulting from low migration among hot spring “islands” in Yellowstone National Park. Patterns of genomic variation indicate that differing geological contexts result in the elimination or preservation of diversity among differentiated populations. We observe multiple deletions associated with a large genomic island rich in glycosyltransferases, differential integrations of the *Sulfolobus* turreted icosahedral virus, as well as two different plasmid elements. These data demonstrate that neither rapid dispersal nor lack of mobile genetic elements result in low diversity in the *S. acidocaldarius* genomes. We suggest instead that significant differences in the recent evolutionary history, or the intrinsic evolutionary rates, of sister *Sulfolobus* species result in the relatively low diversity of the *S. acidocaldarius* genome.

## Introduction

Microbial populations range from fully panmictic to highly geographically structured (Martiny et al. 2006). However, the ecological and evolutionary mechanisms that result in these differences are not well understood. Comparative population biology can be used to identify the mechanisms that structure natural populations if their biology and ecology are close enough to isolate their differences. Comparative population biology on closely related species can provide predictive insight into the environmental and evolutionary factors that contribute to the level of endemism and isolation in microbes.

In the model thermoacidophilic crenarchaeon *Sulfolobus islandicus*, previous work showed that geographic isolation among populations occurs on large spatial scales (Whitaker et al. 2003). On a global scale, genomic signatures demonstrate that both the core and variable components of microbial genomes, including mobile genetic elements, have limited migration (Held and Whitaker 2009; Reno et al. 2009). Low migration results in increased genomic diversity between populations that diverge and evolve independently from one another. These data led to the hypothesis that because *S. islandicus* inhabits small, extreme, and isolated habitats that are geographically distant from each other, low migration results in a strong barrier to gene flow (Whitaker and Banfield 2005).

However, recent work on the closely related species *Sulfolobus acidocaldarius* has cast doubt on the hypothesis that restrictive, extreme environments or geographically distant habitats result in migration barriers. Comparative analysis among three genomes of *S. acidocaldarius* revealed that these genomes, isolated from geographically distant thermal areas in the Norris Geyser Basin in Yellowstone National Park, the Jigokudani thermal field on the island of Hokkaido, Japan and a self-heating mining waste near Ronneburg Germany, were nearly identical (Mao and Grogan 2012). Mao and Grogan (2012) argued that the unusually high conservation among geographically disparate *S. acidocaldarius* strains resulted from wide-scale propagule dispersal, enabling rapid, global-scale gene flow and reducing variation among populations. Because *S. islandicus* and *S. acidocaldarius* are quite similar biologically in the lab and inhabit similar environments, the authors suggested that the stark difference between these species in their population structure and diversity must result not from limited dispersal, but from differences in their ability to colonize and replace local populations (Mao and Grogan 2012). Based on the relative paucity of viral genes in the *S. acidocaldarius* genome (Chen et al. 2005), the authors further suggest that this difference in successful migration may result from *S. acidocaldarius’* resistance to mobile elements, thus preventing the types of selection pressures (such as a host–virus arms race) that create increased diversity within a population and between populations (Held et al. 2013; Childs et al. 2014). An alternative explanation for the homogeneity in *S. acidocaldarius* was that all three strains diverged from a common laboratory culture resulting from contamination (Mao and Grogan 2012), an explanation that could only be tested with independent isolations of multiple strains from separate populations.

To test the hypothesis that high migration and lack of interactions with mobile elements homogenizes the genome of *S. acidocaldarius*, we explored variation in the core and variable genome components of 47 genomes of *S. acidocaldarius* from two hot springs in different geothermal basins in Yellowstone National Park. We used comparative genomics to test for evidence of population structure and to isolate the factors that drive variation in intraspecies diversity.

## Materials and Methods

### Sampling

In September 2012, we collected multiple replicate samples from multiple sites within two hot springs in Yellowstone National Park: GG12 (“Tequila Sunrise”) from Gibbon Geyser Basin, and NG05 (“Lifeboat Spring”) from Norris Geyser Basin. Geochemical data were collected in tandem with the microbiological samples on the first day of sampling (temperature and pH reported in table 1, complete data and methods described in McCleskey et al. 2014). In this study, geochemical measurements were taken near site B at NG05, but previous sampling has confirmed that the chemistry reported at one site is representative of the entire spring (McCleskey et al. 2014). All sampling equipment was ethanol-sterilized. Each bottle was rinsed three times with spring water before sample collection. We sampled turbid water near the bottom of the spring, which is thought to be preferred *Sulfolobus* habitat. We collected samples from two sites ∼2 m apart around NG05 (NG05A and NG05B, see supplementary fig. S1, Supplementary Material online) on four successive days (C03, C04, C05 and C06). After sampling, we isolated microbial colonies through direct plating on DT media (containing basal salts, trace mineral solution, 0.1% dextrin (wt/vol) and 0.1% NZ-Amine) and 0.65% (wt/vol) Gelrite in an overlay solution of 1× DT media (Whitaker et al. 2003) and 0.4% Gelrite. The plates were incubated at 75°C for 1 or 2 weeks or until individual colonies appeared. We subjected each isolate to three rounds of purification on plates, then grew them in liquid phase (1× DT media) for DNA extraction. DNA was extracted using a Qiagen DNeasy Blood and Tissue kit following manufacturer protocols.

**Table 1:** Summary of *Sulfolobus acidocaldarius* Genomes Included in This Analysis

Hot Spring	pH	Temp. (°C)	Latitude, Longitude	Number of Strains	Avg. Genome Size (Mbp)	Avg. Number of ORFs	Percent Core Genes	Number of Clades
GG12	2.61	80.4	44° 41′ 22.5″, 110° 43′ 40.8″	20	2.222 (0.0112)	2412.4 (21.7)	88.12 (0.73)	2
NG05	3.55	58.2	44° 43′ 39.8″, 110° 42′ 49.0″	27	2.215 (0.0213)	2415.6 (31.7)	88.17 (1.13)	5

Note.—Standard deviations are included in parentheses after averages.

### Genome Sequencing, Assembly, and Annotation

Ninety-six isolate genomes were sequenced on an Illumina HiSeq 2000 on one lane for 101 cycles at the University of Illinois Core Sequencing Facility. Sequencing yielded an average of 2,542,340 paired-end reads per sample. We assembled each of the genomes using the a5 pipeline (Tritt et al. 2012) using default parameters. Of the 96 sequenced genomes, the 16S rRNA of 41 genomes matched *S. islandicus* and the remainder matched *S. acidocaldarius*. We selected all *S. acidocaldarius* genomes with greater than 1 million reads to ensure robust assembly with high coverage, resulting in 47 genomes for this analysis.

Assembly of the *S. acidocaldarius* genomes yielded between 1 and 57 scaffolds per genome after assembly (supplementary table S1, Supplementary Material online). The resulting scaffolds were contiguated into a single sequence using abacas 1.3.1 (Assefa et al. 2009), using the single scaffold assemblies as reference, using default parameters with the nucmer flag for mapping. To ensure proper contiguation, reads were mapped back to the assemblies and visualized with bwa and samtools (Li et al. 2009) using default parameters. The contiguated scaffolds were manually checked and rearranged if necessary with Artemis (Rutherford et al. 2000). We verified and corrected all of the assembled sequences by mapping raw reads back to the contiguated scaffolds using breseq (Barrick et al. 2009) using default parameters, and corrected all substitutions, insertions or deletions in the assemblies. We used gdtools (part of the breseq package) with default parameters to apply the corrections to the assemblies.

### Clonal Phylogeny

We created a clonal phylogeny using two methods. The first relied on the identification of SNPs in the core genome using breseq (Barrick et al. 2009). We included three previously sequenced strains: *S. acidocaldarius* DSM639, isolated in Yellowstone National Park in 1972 (Brock et al. 1972; Chen et al. 2005), *S. acidocaldarius* N8, isolated from a thermal field in Hokkaido (Kurosawa et al. 1995; Mao and Grogan 2012), and *S. acidocaldarius* Ron 12/I, isolated from a uranium mine in Germany (Fuchs et al. 1995; Mao and Grogan 2012). We mapped the reads of each *S. acidocaldarius* genome against an ancestral reference genome (*S. acidocaldarius* DSM 639) using default parameters to identify all core SNPs, insertions and deletions. This yielded a total of 150 positions containing a SNP, including positions for which only the reference genomes differed from each other (supplementary table S2, Supplementary Material online). We concatenated all SNPs for each genome to create an alignment, and constructed a maximum likelihood clonal phylogeny using RAxML (Stamatakis 2014) with the rapid bootstrapping method using 100 bootstraps and the GTRGAMMA model of protein evolution. We used *S. acidocaldarius* DSM 639 to root the tree because it was the first to be isolated and most closely approximates the ancestral strain. We used SNAP (Korber 2000) using default parameters to calculate the number of synonymous and nonsynonymous polymorphisms among gene clusters that were present in all 50 genomes. We calculated Tajima’s D test statistic with MEGA7.0.21 (Tamura et al. 2011).

We used ClonalFrame to create a second clonal phylogeny (Didelot and Falush 2007). We aligned all 50 genomes using progressiveMauve (Darling et al. 2004) with default parameters. We used stripSubsetLCBs (part of the Mauve software) to extract locally collinear blocks (LCBs) longer than 500 bp that were shared by all 50 genomes. We applied ClonalFrame to this core genome alignment to create a clonal phylogeny, with 30,000 burn-in iterations and 10,000 MCMC iterations after the burn-in. We conducted three independent runs of ClonalFrame, producing three separate phylogenies (supplementary fig. S2, Supplementary Material online). We used the interactive Tree of Life (iTOL) to visualize all phylogenetic trees (Letunic and Bork 2011).

We used the progressiveMauve alignment to identify genomic islands, defined as regions at least 1000 bp long that were missing from at least one genome based on an alignment of all 50 genomes. We verified the presence and absence of every genomic island in every genome by blasting a representative of each genomic island against every assembled genome. We removed all islands that were missing from only a single genome because most appeared to result from misassemblies. Some large regions with variable genome content were split into several islands as a result of the progressiveMauve algorithm. We grouped genomic islands that were contiguous into larger variable genomic regions (see supplementary table S3, Supplementary Material online).

In order to closely characterize deletions in the genomic island enriched in glycosyltransferases, we used bowtie2 (Langmead and Salzberg 2012) with default parameters to map the reads of each genome to a reference island extracted from GG12_C01_01, and plotted the coordinates at which the coverage dropped <10× (indicating absence of this region in the query genome) and the subsequent coordinates at which the coverage increased to 10× (indicating the edge of the missing locus in the query genome).

### Gene Calling and Clustering

We identified and annotated all open reading frames (ORFs) in the assembled *S. acidocaldarius* genomes using the RAST pipeline (Aziz et al. 2008), specifying domain “Archaea” and genetic code 11. For comparison, we also annotated the three previously sequenced *S. acidocaldarius* genomes with the RAST pipeline to ensure consistent gene-calling algorithms. To confirm annotations of ORFs, we used RPSBLAST to compare all ORFs to the COG database (Tatusov et al. 2003). To compare ORFs in the glycosyltransferase genomic island, we clustered all ORFs using the Integrated Toolkit for the Examination of Microbial Pangenomes (ITEP) (Benedict et al. 2014) using a maxbit score of 0.5 and an inflation value of 2.0.

To compare *Sulfolobus* plasmids, we aligned full plasmid sequences in progressiveMauve (Darling et al. 2004) using default parameters, and created a phylogenetic tree in RAxML (Stamatakis 2014) using the rapid bootstrap algorithm with the GAMMA model of rate heterogeneity and 100 bootstraps. We visualized the trees using Dendroscope (Huson and Scornavacca 2012).

### CRISPR Analysis

CRISPRs were identified in all genomes using the command-line version of the CRISPR Recognition Tool (CRT) (Bland et al. 2007) using default parameters. To compare CRISPRs among *S. acidocaldarius* strains, we conducted an all-v-all BLASTN of every CRISPR spacer against all others within our dataset using a word size of 7, and with dust filtering turned off. We considered a spacer “match” to have an e-value of 0.001 or less. We used the same BLASTN parameters to search for spacer matches against other genomes.

## Results

### Geochemistry of Sampling Sites

We isolated 27 strains from a set of samples from four locations within NG05 (“Lifeboat Spring”) from Norris Geyser Basin, and 20 genomes from a second sample a week later from a second hot spring called GG12 (“Tequila Sunrise”) from the Gibbon Geyser Basin, yielding a total of 47 genomes of *S. acidocaldarius*. Both GG12 and NG05 are acid-sulfate springs characterized by hot gas discharge and chemistry content consistent with mixing of meteoric surface and/or shallow groundwater with deeper, geothermal water (complete dataset can be found in McCleskey et al. 2014). The pH values at the time of sampling were between 2.5 and 3.7, with elevated sulfate concentrations consistent with sulfuric acid generation from H_2_S oxidation (Nordstrom et al. 2009). At the time of sampling, GG12 was hotter and more acidic than NG05 (table 1). GG12 is ∼30 cm in diameter and located in the main drainage of the thermal area near the base of a steep hillside in Gibbon Geyser Basin. NG05 is ∼6 m in diameter and is located in a geothermal plain in the Ragged Hills area of Norris Geyser Basin. Although we did not directly observe precipitation-driven flushing events in Gibbon Geyser Basin from 2010 to 2012, we have observed substantial surface flushing events in other acid-sulfate springs of similar and larger size in the Nymph Lake Area of Yellowstone National Park, and hypothesize that flushing events are important in the small Gibbon Geyser springs as well. NG05 exhibits geochemical variability in mixing of deep geothermal and shallow subsurface waters, but it is not driven by surface events, likely due to its relatively large size.

The *S. acidocaldarius* genomes ranged in size from 2.18 to 2.24 Mbp, with greater variation in genome size among the NG05 genomes compared with the GG12 genomes (table 1 and supplementary table S1, Supplementary Material online). We identified core positions as those shared among all strains, and variable genes as those that were absent in at least one genome. In the core genome, we observed a total of 119 SNPs, and 21 insertions, 10 deletions, and 4 substitutions of 1–10 nucleotides among all 47 newly sequenced *S. acidocaldarius* genomes isolated from Yellowstone in 2012 (table 2 and supplementary table S2, Supplementary Material online) in comparison to the previously sequenced reference genome *S. acidocaldarius* DSM639 (Brock et al. 1972; Chen et al. 2005). These SNPs were distributed across the chromosome (fig. 2 and supplementary table S2, Supplementary Material online). Thirty-one SNPs, 9 deletions and 6 insertions were identical in the newly sequenced genomes but diverged from the reference genome.

**Table 2:** Pairwise Average Nucleotide Divergence and Polymorphisms in *Sulfolobus acidocaldarius* and *S. islandicus* among Core Genomes

Genome Comparisons	Average Pairwise Nucleotide Divergence	Number of SNPs	Species	Sampling Locations	Reference
Comparisons among all 47 *S. acidocaldarius* strains from YNP 2012	5.91 × 10^−5^	119	*S. acidocaldarius*	YNP (2012)	This study
DSM 639 vs. N8	3.70 × 10^−6^	9	*S. acidocaldarius*	YNP (1972) vs. Japan	Mao and Grogan (2012)
N8 vs. Ron12/I	1.30 × 10^−5^	30	*S. acidocaldarius*	Japan vs. Germany	Mao and Grogan (2012)
Ron12/I vs. DSM 639	1.66 × 10^−5^	38	*S. acidocaldarius*	Germany vs. YNP (1972)	Mao and Grogan (2012)
Comparisons among Yellowstone strains	2.60 × 10^−3^		*S. islandicus*	YNP	Reno et al. (2009)
Comparisons between North American strains	4.60 × 10^−3^		*S. islandicus*	YNP vs. LNP	Reno et al. (2009)
Comparisons between each North American and Mutnovsky strain	1.11 × 10^−2^		*S. islandicus*	YNP/LNP vs. Mutnovsky	Reno et al. (2009)

Note.—We identified SNPs using breseq (see “Materials and Methods” section) and calculated average nucleotide divergence as the number of polymorphisms divided by the core genome size. YNP, Yellowstone National Park; LNP, Lassen National Park.

### Core Genome

The pairwise nucleotide divergences for the 47 *S. acidocaldarius* core genomes were extremely low, at 5.91×10^−5^ (table 2). This is on the same scale as previously observed in *S. acidocaldarius* strains from global populations, with an average nucleotide divergence of 1.1×10^−5^ (Mao and Grogan 2012). In contrast, average nucleotide divergences for *S. islandicus* strains isolated from Yellowstone National Park were two orders of magnitude higher (Reno et al. 2009) (table 2).

**Fig. 1.—:**
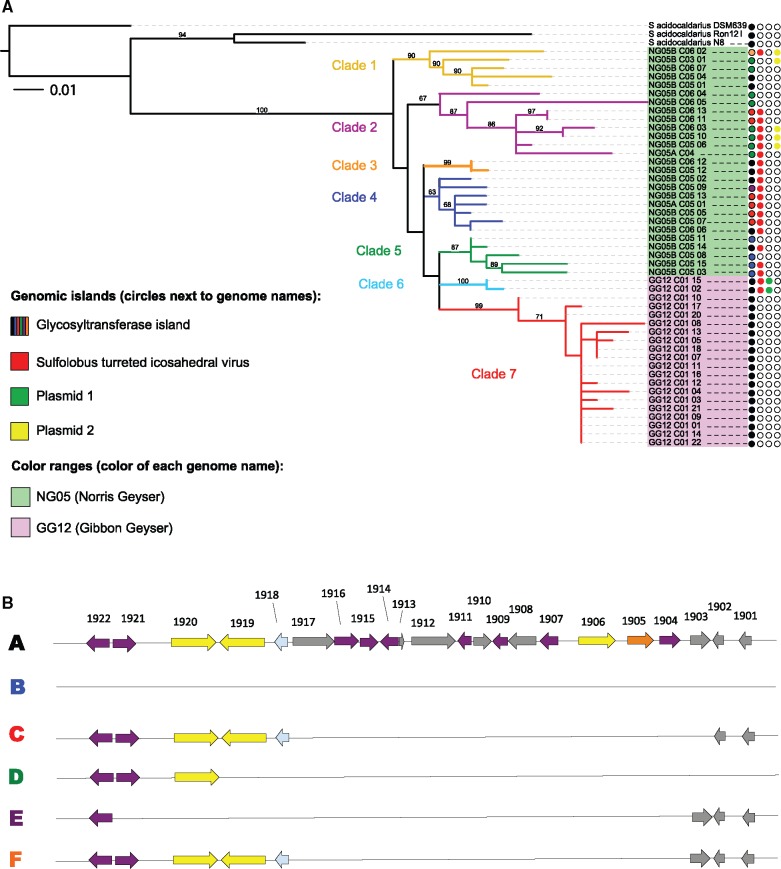
Clonal phylogeny with large genomic insertions and deletions. (*A*) Phylogenetic tree constructed by concatenating SNPs identified in the core genome. The colored lines of the tree indicate each of the five clades we identified among the *Sulfolobus acidocaldarius* genomes isolated in 2012. Leaves are color-coded according to the hot spring from which they were isolated. Circles to the right of leaves indicate the presence or absence of genomic islands. First column; glycosyltransferase island; second column, STIV; third and fourth columns, plasmids 1 and 2, respectively. The colors in the glycosyltransferase island correspond to the deletion patterns shown in part (*B*). Trees were visualized using the interactive tree of life (ITOL) program (itol.embl.de). (*B*) Complete open reading frames found in the large genomic island encoding glycosyltransferases. We observed six different deletion patterns in this genomic island; the letters to the left indicate the designated deletion pattern. These correspond to the deletion pattern letters shown in supplementary table S7, Supplementary Material online. The numbers at the top indicate the ORF number of the ORF in *S. acidocaldarius* DSM639, with “Saci_” preceding the number in the ORF label. These genes are described in supplementary table S6, Supplementary Material online. Purple: glycosyltransferases, yellow: membrane proteins, light blue: methyltransferases, green: cell envelope, surface polysaccharides and lipopolysaccharides, orange: metabolite transport-related protein, gray: hypothetical protein.

A phylogeny based on identification of SNPs in the core genome clearly differentiates the 47 newly sequenced *S. acidocaldarius* strains from the three previously sequenced genomes (Mao and Grogan 2012). The tree splits the genomes into seven well-supported clades, whose relationship to one another is unresolved (fig. 1). Strains from NG05 form five monophyletic groups (clades 1–5) that each contain strains from different samples at different times within this large spring. The GG12 genomes form two monophyletic groups, clades 6 and 7 (fig. 1). A clonal phylogeny based on both SNPs and indels <10 bp had the same topology as the SNP phylogeny (supplementary fig. S3, Supplementary Material online). This indicates that the populations in both GG12 and NG05 consist of several genotypes that coexist, and that the populations that persist within in these two hot springs are differentiated from one another.

The three clonal phylogenies created using ClonalFrame resulted in three slightly different trees (supplementary fig. S2, Supplementary Material online). Although the placement of the internal branches varied, seven distinct clades emerged in all three of the ClonalFrame trees. These seven clades were almost identical to the seven clades we observed in figure 1, with the exception of NG05B_C05_02, which moved from clade 1 in the SNP tree to clade 3 in the ClonalFrame trees.

In addition to the phylogenetic analysis, we identified differentiation among isolated populations by the presence of private alleles that were uniquely found in one clade and in more than one strain. The informative positions (SNPs found in more than one genome) show that the majority of SNPs (36 out of 45 biallelic positions) are private alleles. Clade 7, which contains almost all of the GG strains, has the largest number of unique and fixed or nearly fixed SNPs (6). Two additional strains from GG12 in Clade 6 maintain 3 unique SNPs that differentiate them from the other GG12 strains.

We observed more singleton SNPs (found in only one genome) among the NG05 genomes than the GG12 genomes. The NG05 genomes all together were differentiated by 0–10 singletons per genome. We observed no evidence for separation of clades by geographic location or sampling time within the hot spring (fig. 1). In contrast, the GG12 genomes all together were differentiated by only 0–4 singletons per genome. The Tajima test statistic (D) for each of the clades was lowest for clade 7 (supplementary table S4, Supplementary Material online). The abundance of fixed SNPs and the relative absence of singleton SNPs within clade 7 is consistent with the hypothesis that these genomes experienced a recent bottleneck or colonization event.

**Fig. 2.—:**
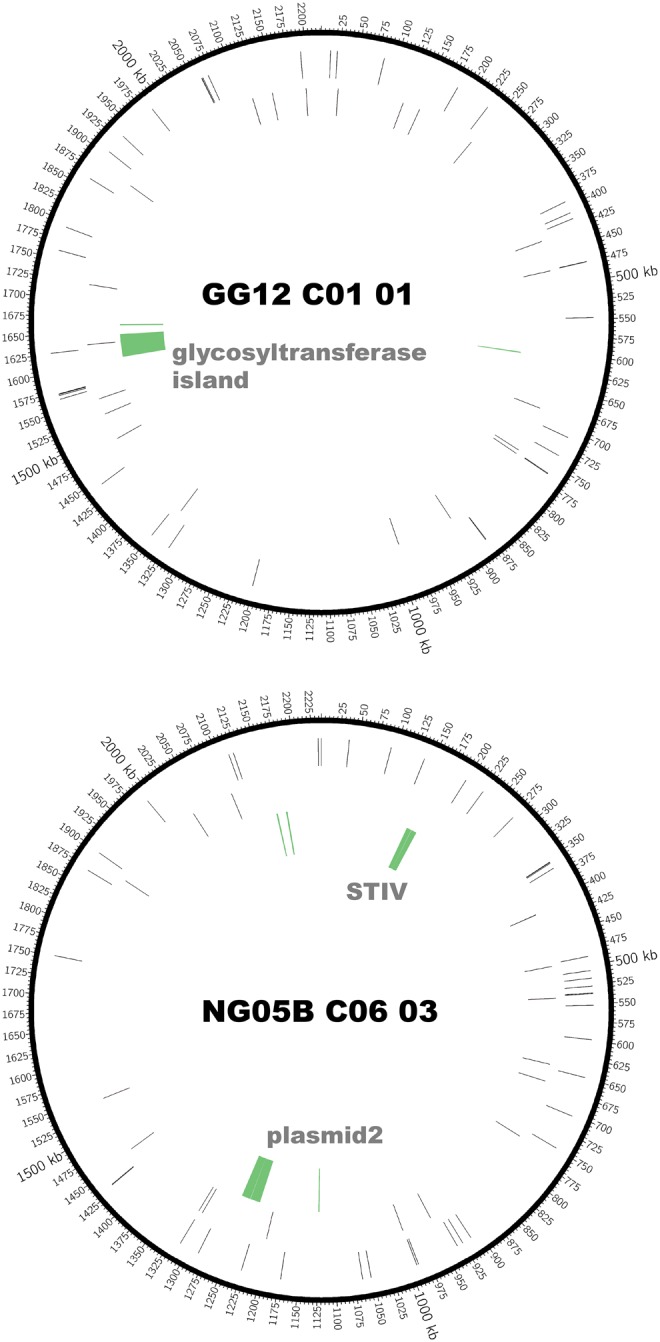
Map of chromosome features for genome GG12_C01_01 and NG05B_C06_03. *Outer ring*: location of single nucleotide polymorphisms (SNPs) in the core genome. *Middle ring*: location of small insertions and deletions (indels) in the core genome. *Inner ring*: large insertions (>100 bp) relative to *Sulfolobus acidocaldarius* DSM 639. The width of the green line represents the size of the insertion, with a minimum line width of 0.25 pt.

To search for signals of selection among clades, we assessed whether the SNPs in the core genome changed the coding sequence of specific proteins (supplementary table S5, Supplementary Material online). Among core genes overall, there were ∼3.6×10^5^ potential synonymous sites and 1.3×10^6^ potential nonsynonymous sites. We observed 53 nonsynonymous SNPs and 41 synonymous SNPs in the core genome, yielding a dn/ds ratio of 0.360 overall, which indicates recent introduction of mutations that have not yet been cleared by selection. With so few mutations, the chance of multiple mutations occurring in a single *S. acidocaldarius* gene without selection is low. We identified three genes with multiple substitutions that were fixed between our 47 strains and the reference strain (table 3). We found three additional genes with multiple nonsynonymous substitutions and indels that vary within our population relative to the references. As shown in table 3, these six genes are predicted to encode two membrane proteins and a helicase and vary within either the NG or the GG populations. We suggest this may be due to interactions with viruses in each environment, as mutations in a homolog of *Saci_2317* have been shown to confer resistance to the lytic virus SIRV (Deng et al. 2014). With the exception of one allele for *Saci_1274*, which was fixed in clade 2, none of these loci we hypothesize to be under selection is uniquely associated with a clade or responsible for its differentiation. In addition, we identified one gene, *Saci_2099*, with 10 synonymous mutations in a gene with the putative annotation of encoding a solute binding protein. This gene was likely acquired by horizontal gene transfer from outside of the population into the strains from the NG population. Besides these focal genes, the locations of the SNPs and indels did not appear to occur in specific regions of the genome, nor did the SNPs differentiating genomes appear to occur in localized regions (fig. 2).

**Table 3:** Loci with Multiple Mutations

	No. Synonymous SNPs	No. Nonsynonymous SNPs	No. Deletions	Annotations	Notes
Deviations from DSM639					
*Saci_0266*	0	3	0	Phosphate transport regulator (distant homolog of PhoU)	
*Saci_0783*	0	3	0	NAD (FAD)-dependent dehydrogenase	
*Saci_1127*	0	0	0	Amino acid permease	
Variation within YNP 2012 Population					
*Saci_1274*	0	2	0	Uncharacterized membrane protein, required for N-linked glycosylation	Both variable within the NG population
*Saci_1320*	0	7 (one nonsense)	2	ATP dependent helicase	All singletons in the NG population
*Saci_2317*	0	2	0	Type IV secretory pathway archaeal flagella biosynthesis	Three alleles in the GG population

Note.—All genes are listed as annotated in *Sulfolobus acidocaldarius* DSM 639.

### Variable Genome

We identified four large (20 kb or larger) variable genomic regions that vary among the genomes of all 50 *S. acidocaldarius* strains, plus four smaller genomic islands of 1 kb or larger (supplementary table S3, Supplementary Material online). On an average, these variable regions constituted ∼2.5% of the genome across all *S. acidocaldarius* genomes (supplementary table S1, Supplementary Material online). The four large genomic regions encoded a series of genes related to glycosyltransferases and membrane proteins, a *Sulfolobus* turreted icosahedral virus (STIV), and two plasmids. To better understand the population dynamics of these variable gene islands, we mapped them onto the core genome phylogeny in figure 1. This shows different dynamics for different variable loci.

Among the large variable genomic islands was a 33-kb region that contained many GT1 family glycosyltransferases, as well as predicted membrane proteins, methyltransferases, and glycosyltransferase family 2 (figs. 1 and 2; supplementary table S6, Supplementary Material online). This region is located between 1603770 and 1645021 in GG12_C01_01, and between 1694040 and 1732388 in *S. acidocaldarius* DSM 639. A list of genes encoded on the genomic island is presented in supplementary table S6, Supplementary Material online. All of the genomes from GG12 had full coverage of the genomic island; in contrast, the genomes from NG05 lacked different regions of the island or were missing the island entirely (fig. 1 and supplementary table S7, Supplementary Material online). Based on the presence of this locus in previously sequenced genomes and the differential distribution of gene presence and absence, we infer that multiple gene losses are responsible for the variation in NG05 (fig. 1). We observed six different deletions within this locus. The smallest deletion (C) is shared among clades 2 and 4 within NG05, suggesting it occurred and persisted in the population before the clades diverged (supplementary table S7, Supplementary Material online). Clades 1 and 2 share an additional, possibly subsequent deletion (D). Additional unique deletions are found in all but one of the strains in clade 5 (B) and individually in strain NG05B_ C05_09 (E) and NG05B_C06_02 (F). The maintenance of variation in this locus and the parallel deletions suggest that diversifying selection is acting on this locus within this population.

The second genomic island we observed encoded a set of 30 genes that match the *Sulfolobus* turreted icosahedral virus (STIV). This integrated virus was encoded almost exclusively in NG05 genomes, but was also observed in GG12_C01_02 and GG12_C01_15, which were distinct from the other GG12 genomes in the clonal phylogeny (fig. 1). Prior to this observation, the only known host for STIV was a derived variant of *S. solfataricus* (Rice et al. 2004). Its native host is unknown. The STIV genomes found in these *S. acidocaldarius* isolates are identical to each other. They are similar to the STIV genomes that have been sequenced previously (Rice et al. 2004), with an average of 75.4% and 67.5% amino acid identity to STIV1 and STIV2, respectively (supplementary table S8, Supplementary Material online). The virus is integrated into an ORF encoding an ABC-type transporter.

Finally, we identified two different plasmids integrated into the genomes of several *S. acidocaldarius* strains (figs. 1 and 2). These plasmids are very different from one another, sharing <10% of sequence. Plasmid 1, ∼30 kb in length, was found in the same genome context in GG12_C01_02 (723009–752610) and GG12_C01_15 (1301442–1326211), in clade 6 in the clonal phylogeny (fig. 1). These plasmids are nearly identical, indicating a single insertion event occurred in this lineage. This plasmid shares 83% nucleotide identity across 46% of their length with an integrated plasmid in the strain of *S. islandicus* M.16.4 isolated from Kamchatka (Schleper et al. 1995) and 82% identity across 43% of their length to the conjugative plasmid pNOB8 isolated from *Sulfolobus* strain NOB8H2 from Japan (She et al. 1998). Interestingly, although it shares shorter length (only 7% coverage) it shares higher identity with an integrated plasmid in *S. islandicus* strain Y.N.15.51 from the Norris Geyser Basin in Yellowstone National Park. Plasmid 2, ∼35 kb in length, was nearly identical in strains NG05B_C06_02 (1313858–1349698) and NG05B_C03_01 (1392216–1426722) in clade 1, and NG05B_C05_06 (1388980–1425097), NG05B_C05_10 (714330–748821), and NG0B_C06_03 (1229613–1263995) in clade 2, and found in the same genome context for all genomes. This suggests either two independent acquisitions or a single gain and two losses of this plasmid in the history of these lineages. Plasmid 2 shares 96% identity over 32% and 51% of the length with *S. islandicus* strains Y.G.57.14 and L.D.8.5 from Yellowstone’s Gibbon Geyser Basin and Lassen National Park, respectively. It shares 94% identity over 24% of its length with plasmid pYN01 from *S. islandicus* strain Y.N.15.51. The similarity between portions of plasmids from different *Sulfolobus* species suggests that the *Sulfolobus* plasmids have a broad host range. However, with so few examples, it is not possible to determine the population biology and differentiation among genomic regions from these data. While plasmid 1 contains section A, which encodes conjugation genes; plasmid 2 contained only one ORF from this conserved region shared among conjugative plasmids (Greve et al. 2004). Conserved regions B and C were missing from both *S. acidocaldarius* plasmids. All of the remaining genes on these plasmids are annotated as hypothetical.

#### Insertion Sequences

We identified only seven insertion sequence (IS) elements in each genome, including five transposases and two integrases. In contrast, *S. islandicus* genomes isolated from Yellowstone have much higher abundances of IS elements: for example, *S. islandicus* Y.G.47.14 contains 118 IS elements, and *S. islandicus* Y.N.15.51 contains 32 (Reno et al. 2009). The IS elements we identified in *S. acidocaldarius* have significant matches (e-value <10^−5^) to each of the *S. islandicus* genomes available in the NCBI database. Thus, although the IS elements between *S. islandicus* and *S. acidocaldarius* from Yellowstone are similar to each other, their relative abundances in each species vary drastically.

The original *S. acidocaldarius* DSM 639 genome contained four IS elements, designated SA1–SA4 (Chen et al. 2005). Of these, SA1 and SA3 occurred in all 50 of the genomes analyzed here. SA2 was present only in the three previously sequenced genomes. SA4 was present in all of the genomes except for NG05B_C05_06. All of the shared original IS elements occur in the same genomic context.

#### *CRISPR Loci Show Evidence of Mobile Elements Shared between* Sulfolobus *Species*

CRISPR spacers are effectively libraries of previous viral infection, and can therefore act as useful tools for observing patterns of differentiation among closely related strains (Andersson and Banfield 2008; Held and Whitaker 2009; Held et al. 2010; Rho et al. 2012; Vestergaard et al. 2014). We identified four CRISPR loci in the genomes of each of the 47 *S. acidocaldarius* strains. All 4 CRISPR loci among all 50 strains were identical in terms of spacer content and order including the three previously sequenced genomes, despite having been isolated from distant time points and geographic locations. All *S. acidocaldarius* genomes possess core CRISPR-associated (*cas*) genes *cas1, cas2, cas3, cas4, cas6, csm3*, and *csm6*. This includes type 1A CRISPR/Cas systems relative to the new classification (Makarova et al. 2011; Vestergaard et al. 2014).

In comparing CRISPR spacer sequences against the *S. acidocaldarius* genome, we identified self-matches including the integrated STIV, plasmids and the regions of deletion within the glycosyltransferase-rich gene island. These data indicate either that the CRISPR system in *S. acidocaldarius* is inactivated, that there is anti-CRISPR activity that is preventing self-targeting, or that the crRNA is expressed at a low level or not expressed at all. In addition, we compared all *S. acidocaldarius* CRISPR spacers against all available *Sulfolobus* viral genomes and *S. islandicus* genomes. Among *Sulfolobus* viruses, only *S. islandicus* rod-shaped viruses (SIRV) 1 and 2 (NC_004086 and NC_004087) had a match to the *S. acidocaldarius* CRISPR spacers (supplementary table S9, Supplementary Material online). We observed several matches between the *S. acidocaldarius* spacers and *S. islandicus* genomes, most of which seemed to match regions on the genome encoding plasmids and transposase/IS elements (supplementary table S9, Supplementary Material online) as well as CRISPR spacers. CRISPR spacers from *S. islandicus* HVE10.4, L.D.8.5, L.S.2.15, LAL14.1, M.14.25, M.16.27, M.16.4, REY15A, Y.G.57.14, and Y.N.15.51 had matches to the *S. acidocaldarius* integrated STIV, indicating that this virus is similar to STIV viruses that have infected *S. islandicus* in the past. Finally, *S. islandicus* spacers were also found to match plasmids integrated in *S. acidocaldarius*. These CRISPR matches indicate that the *S. acidocaldarius* plasmids are similar to other plasmids that have infected *S. islandicus* in the past. Overall, the cross-matches between *S. acidocaldarius* and other *Sulfolobus* CRISPR spacers, transposases, plasmids, and viruses indicate that mobile elements can cross the species boundary within the *Sulfolobus* genus, and that *S. acidocaldarius* is not entirely resistant to infection by mobile elements.

## Discussion

### Highly Conserved the Core Genome Reveals Biogeographic Patterns

In contrast to previous work describing nearly identical core genomes from three globally distributed *S. acidocaldarius* strains, this comparison of 47 genomes revealed population structure within and between different hot springs in Yellowstone National Park. In particular, we observe differentiation among several clades, each containing only members from specific hot springs, indicating that there is biogeographic differentiation among populations from two different geothermal regions within Yellowstone National Park (see fig. 1). These results indicate that there is low migration among hot springs, resulting in decreased rates of recombination and gene flow between populations. Despite the fact that each of our strains was isolated on the same media in the same way, we found evidence of more genomic variation than had previously been reported for this species (Mao and Grogan 2012) on much smaller spatial and temporal scales, and a clear pattern of differentiation among hot springs. While cultivation bias could select for specific sets of strains, we would expect cultivation bias to select for more homogeneity among strains rather than variation.

Variation within the core genome provides a snapshot that we can use to derive previous events in the history of the populations. Patterns of core SNPs suggest that compared with the NG05 genomes, the GG12 genomes had fewer singleton SNPs, which represent SNPs that have recently emerged and have not yet become fixed in the population, and therefore the GG12 populations appear to be more recent. The dn/ds ratio of the core genome was not indicative of strong selection. One hypothesis to explain these patterns is that differences in the environmental stability of these hot springs lead to variation in the intraspecies diversity of *S. acidocaldarius.* GG12 is a small hot spring in a large drainage at the base of a steep hill, and is likely subject to frequent precipitation-driven flushing events. Flushing events can lead to a rapid change in environmental conditions and may also flush or dilute the microbial population in the hot spring, which can result in bottleneck events that eliminate genomic variation from the population. In this scenario, genetic drift could act as an important driver in the evolution of microbial genomes over time. The absence of a strong signal for selection supports this hypothesis. We note that the warmer temperature and lower pH of GG12 were closer to optimal *Sulfolobus* culturing conditions compared with NG05, raising the question of whether this affected the population of *Sulfolobus* in each spring. However, unfavorable growth conditions in NG05 might impart a strong selective pressure or create a bottleneck for growth, which is the opposite of what we observed in NG05. It is also possible that there could be differences in the rates at which new mutations are introduced between the two hot springs (higher in lower temperatures), though this is less likely.

In contrast to GG12, the NG05 hot spring is larger in diameter, has higher surface volume, and is located on a plain. Because of its geographic context, NG05 may experience fewer high-volume flushing events than GG12, therefore increasing the environmental stability of this spring. As a result, microbial populations are exposed to stable conditions and fewer flushing or dilution events, allowing more time for mutations to accumulate and spread, thus supporting the proliferation of greater intraspecies genomic diversity (Campbell et al. 2017). We observed multiple clades within NG05 that did not correspond to sample location or time, suggesting that these clades are differentiated due to barriers to recombination as seen in *S. islandicus* previously (Cadillo-Quiroz et al. 2012) or ecological heterogeneity that we have not measured. Previous sampling efforts have suggested that the geochemical composition in the hot spring is relatively homogenous in bulk samples (McCleskey et al. 2014), but microscale gradients or heterogeneity within sediments or particles have not been evaluated, and may create barriers to recombination. We observed some evidence of recombination between Clades 1 and 2 and between Clades 1 and 5, indicating that any barriers to recombination are incomplete. However, the extremely low number of SNPs in the core genome in *S. acidocaldarius* makes it difficult to identify definitive cases of recombination, and thus the causes for sympatric differentiation in NG05 are inconclusive and do not point to any obvious source of ecological selection. The absence of strong signals of selection and the differences in genomic diversity between the hot springs suggest that environmental stability, rather than geochemistry itself, can be an important driver of genomic diversity.

Despite this new evidence for biogeographic differentiation in *S. acidocaldarius*, the high conservation of the *S. acidocaldarius* core genome remains a stark contrast to the much lower core genome conservation of *S. islandicus*, which inhabits the same geochemical environments. The reason for the differences in genome diversity between *S. islandicus* and *S. acidocaldarius* is unclear: either genomic variation is introduced very rarely in the *S. acidocaldarius* genome, or variation was removed very recently through a selective sweep or genetic bottleneck. The differences between these species suggest that the *S. acidocaldarius* population emerged more recently than the *S. islandicus* populations we have studied, or it has experienced a more recent purifying event; however, this hypothesis would require that all *S. acidocaldarius* genomes studied to date, including those examined prior to this study (Mao and Grogan 2012), were subjected to a purifying event in the relatively recent past compared with the *S. islandicus* genomes that have been studied*.* Alternatively, it is possible that there are differences in mechanisms of mutation, repair, or recombination that result in a more rapid generation of variation in *S. islandicus* than *S. acidocaldarius* in nature, despite similar mutation rates observed in the laboratory (Grogan et al. 2001; Blount and Grogan 2005). Previous work has suggested that IS elements can generate genome instability in other *Sulfolobus* strains (Brügger et al. 2004), and *S. acidocaldarius* genomes possess very few IS elements. However, the Kamchatka population of *S. islandicus* also has very few IS elements (Guo et al. 2011; Cadillo-Quiroz et al. 2012), yet it has accumulated orders of magnitude more variation in its core genome. Therefore it seems unlikely that the paucity of IS elements is the sole reason for the high stability of the *S. acidocaldarius* genome. Horizontal gene transfer of core genomic regions may lead to gene conversions, and if horizontal gene transfer and gene conversion are more frequent in *S. acidocaldarius* compared with *S. islandicus*, this may be a possible explanation for the difference in core genome conservation between the two species. However, our data do not show variation in the density of SNPs across the genomes, and therefore we do not currently have evidence supporting the hypothesis that recombination or horizontal gene transfer is more common in *S. acidocladarius* compared with *S. islandicus*.

### Variation in the Variable Genome Indicates Susceptibility to Mobile Elements

While the core genome of *S. acidocaldarius* appears to be highly conserved, we observed several genomic islands that define the variable genome of *S. acidocaldarius* resulting from genome deletions and acquisitions of new genes from mobile genetic elements. The largest deletions observed were in a region of the genome rich in glycosyltransferases. This island was present in all of the GG12 genomes but only a few NG05 genomes. Genomic islands encoding glycosyltransferase have been previously observed to be highly variable in *S. islandicus* (Reno et al. 2009; Guo et al. 2011; Cadillo-Quiroz et al. 2012) as well as other species (Cuadros-Orellana et al. 2007; Peña et al. 2010; Grote et al. 2012; Kashtan et al. 2014; Meyer and Huber 2014). Previous work has suggested that this cluster appears to be involved in the formation of the EPS matrix in *S. acidocaldarius* (Orell et al. 2013). Variation in the presence or absence of a glycosyltransferase cluster may reflect evolutionary pressure to alter the structure or composition of the EPS matrix, which forms the outermost protective barrier for the cell and is involved in biofilm formation.

It was previously suggested that *S. acidocaldarius* may be highly conserved because it is uniquely resistant to foreign genetic elements (Mao and Grogan 2012). The presence of IS elements, an integrated STIV, and two new plasmids in these newly isolated *S. acidocaldarius* genomes indicates that foreign genetic elements can and do invade the genome of *S. acidocaldarius*. Previous work has indicated that *S. acidocaldarius* contains restriction-modification systems absent from other *S. acidocaldarius* strains, suggesting that *S. acidocaldarius* may only be susceptible to mobile elements with specific modifications on their genomes (Chen et al. 2005). The observation that several IS elements in the Yellowstone 2012 genomes are homologous to IS elements present in *S. acidocaldarius* DSM 639, and the observation that the plasmid and integrated provirus are similar across the Yellowstone 2012 strains, supports the possibility that S*. acidocaldarius* is susceptible to specific groups of mobile elements. In contrast, a wide variety of strain-specific mobile elements have been observed on the genome of *S. islandicus* (Reno et al. 2009). Reduced susceptibility to mobile genetic elements in *S. acidocaldarius* would result in higher genome conservation, because mobile genetic elements play important roles in transposition and horizontal gene transfer. Signatures in the CRISPR sequences suggest that *S. acidocaldarius* and *S. islandicus* are susceptible to similar viruses, plasmids and insertion elements. However, the spacers within the CRISPR loci were conserved across all 50 *S. acidocaldarius* genomes. This further supports the hypothesis that *S. acidocaldarius* is less susceptible to mobile elements than *S. islandicus*, which possesses much more variable CRISPR loci (Held and Whitaker 2009; Held et al. 2010, 2013; Guo et al. 2011). Alternatively, it is also possible that the *S. acidocaldarius* CRISPR loci are defective despite being transcriptionally active (Lillestøl et al. 2009). If this is the case, alternative mechanisms such as blocking of phage receptors, restriction/modification systems, or abortive infection systems are more important than the CRISPR/Cas system for providing immunity to foreign genetic material in *S. acidocaldarius*.

### Conclusion

The *S. acidocaldarius* genome is unusual in its high degree of conservation across space and time. Here, in contrast to previous studies suggesting that the highly conserved genome provided support for rapid, global-scale gene flow, we provide definitive evidence for differentiation in this species within and between hot spring environments. We conclude that high conservation in the core genome of *S. acidocaldarius* is not the result of rapid migration, and our data support the hypothesis that the highly structured nature of extreme environments results in differentiated subpopulations linked by limited migration even at relatively small scales. The observation of plasmids, insertion sequences and prophage integrated in the genome provide evidence that the high conservation is not uniquely caused by resistance to foreign genetic elements, though reduced susceptibility to mobile elements may contribute to genome conservation. Moreover, patterns in the core genome suggest that environmental stability has an important impact on intraspecies genomic diversity, and that genetic drift plays an important role in the evolution of microbial populations. Understanding such patterns of microbial evolution, and how these patterns differ between species, provides crucial insight into the eco-evolutionary dynamics that generate and maintain diversity in microbial populations.

## Supplementary Material

Supplementary data are available at *Genome Biology and Evolution* online.

## Supplementary Material

Supplementary figures

Supplementary tables
