# Supplementary material for: Structured Populations of Sulfolobus acidocaldarius with Susceptibility to Mobile Genetic Elements
Source: Genome Biol Evol. 2017 Jun 14;9(6):1699–710. doi: 10.1093/gbe/evx104 (PMC5554439; doi:10.1093/gbe/evx104)
Supplement: Supplementary figures [file evx104_SuppFig.pdf]

A)

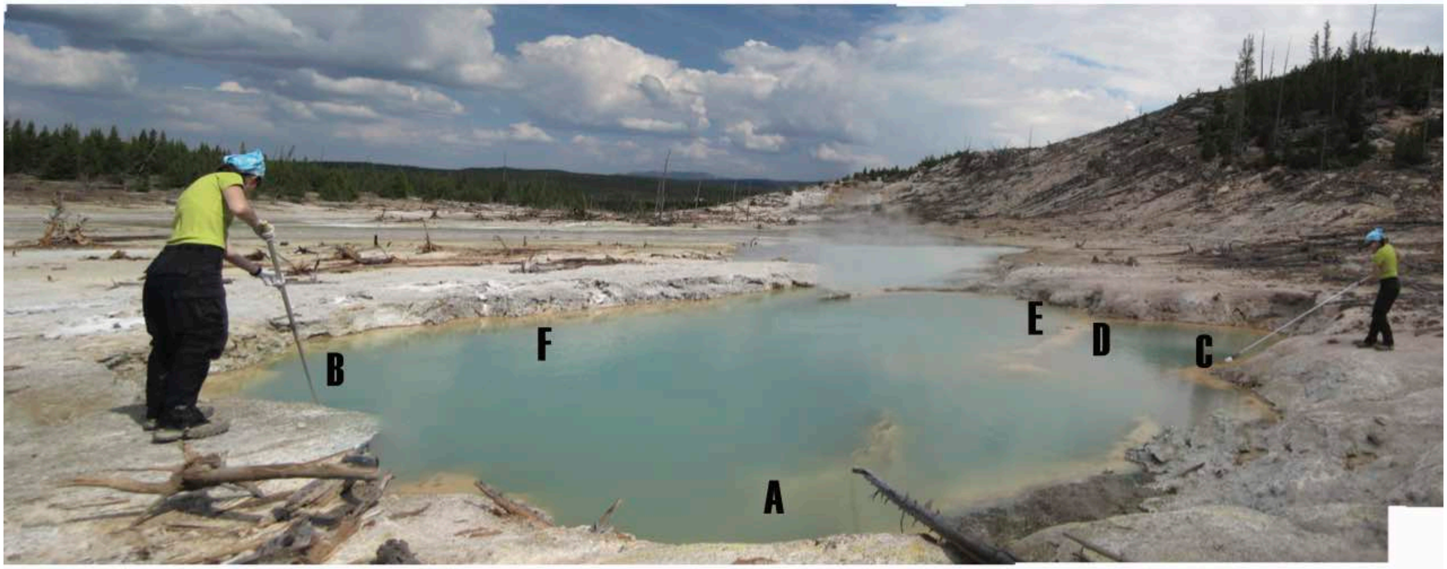

B)

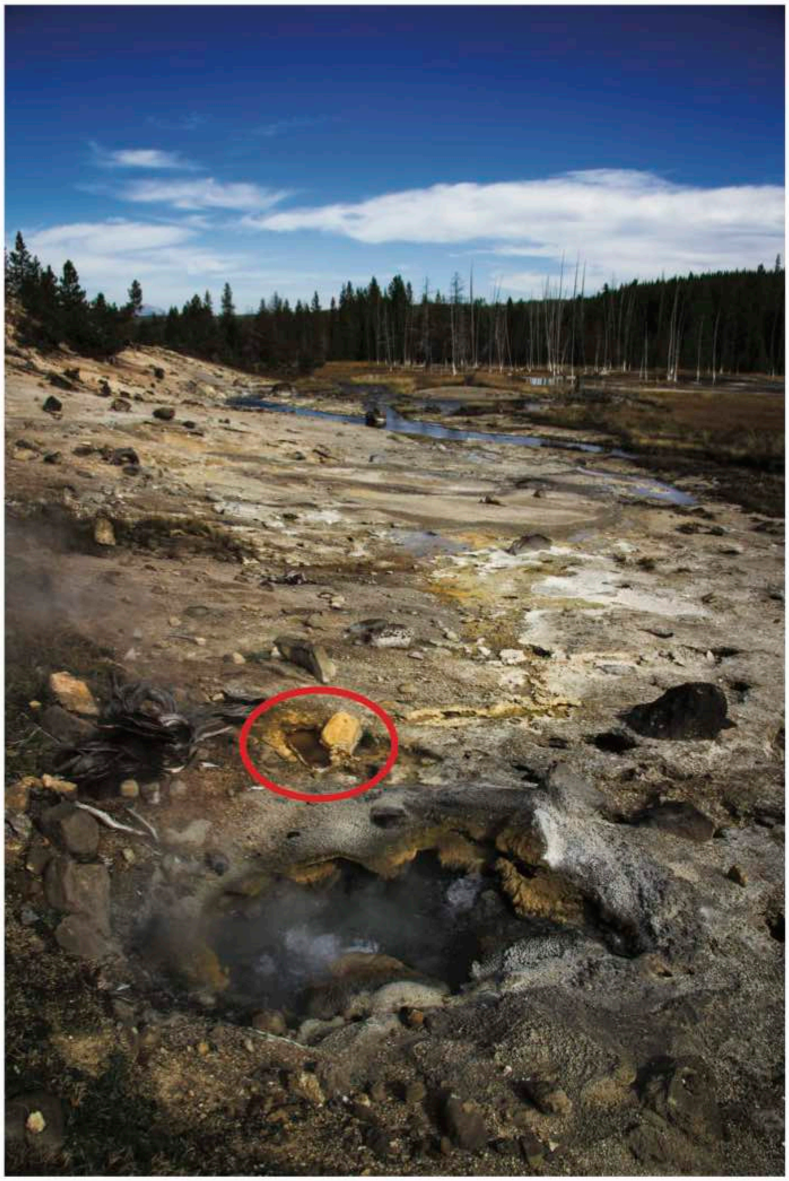

Supplementary Figure 1. Images of sampling sites. A) NG05 hot spring in the Norris Geyser Basin. Sampling sites A-F are labeled on the image. Samples for this study were taken at sites A and B. B) GG12 hot spring in the Norris Geyser Basin. GG12 is the small hot spring circled in red.

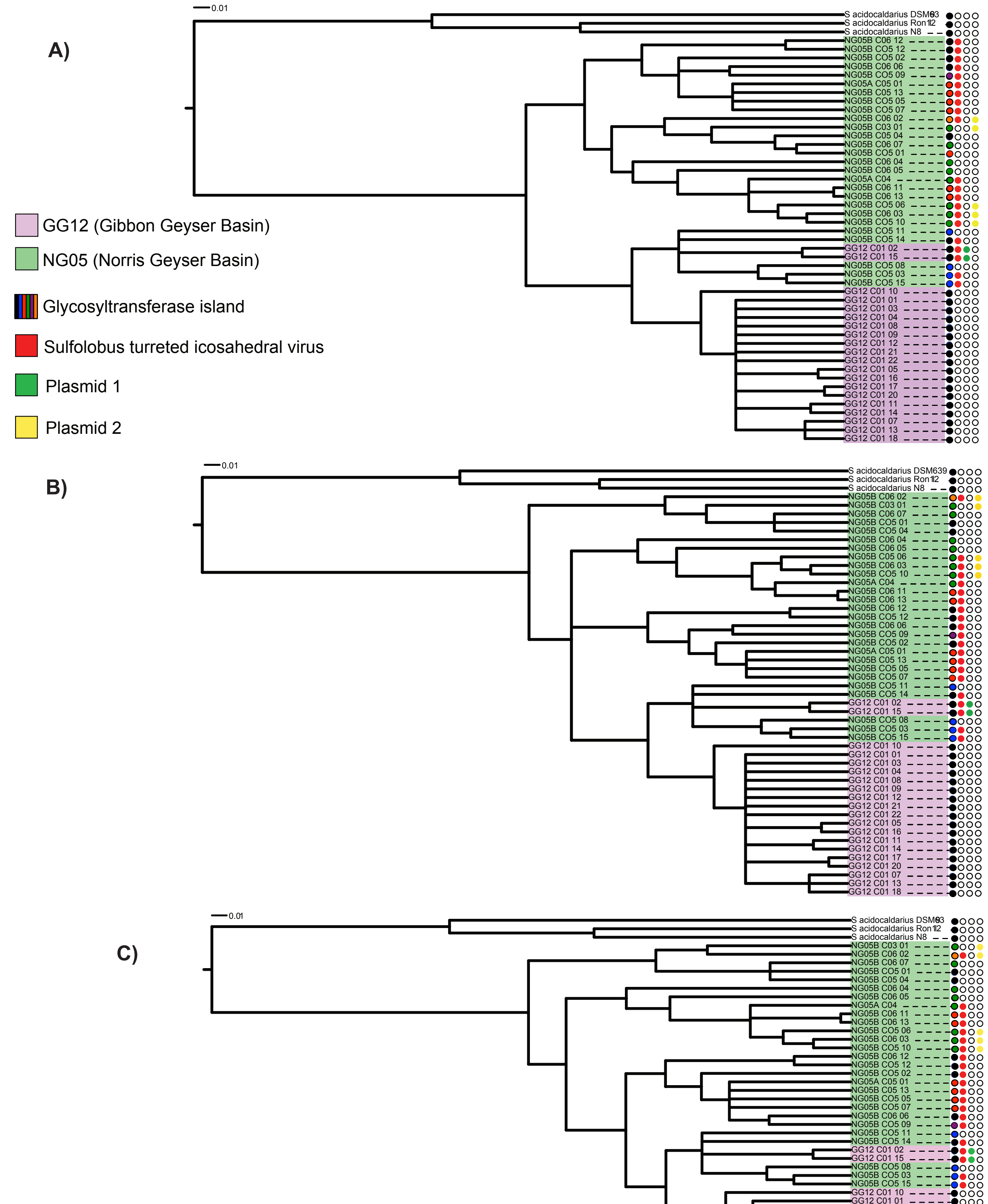

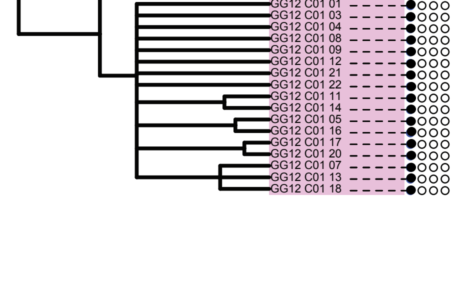

Supplementary Figure 2. Clonal phylogeny inferred from conserved locally collinear blocks. Clonal relationships inferred by ClonalFrame (Didelot & Falush, 2007). Each tree (A, B, and C) represents an independent, parallel ClonalFrame run with identical input parameters. The branch lengths are measured in coalescent units of time, which are equal to the effective population size, Ne, times the duration of a generation (Didelot et al. 2011). Dots to the right of leaves indicate the presence or absence of genomic islands, and are colored as in Figure 1. Leaves are color-coded according to the hot spring from which they were isolated. Trees were visualized using the Interactive Tree of Life (ITOL) program (itol.embl.de).

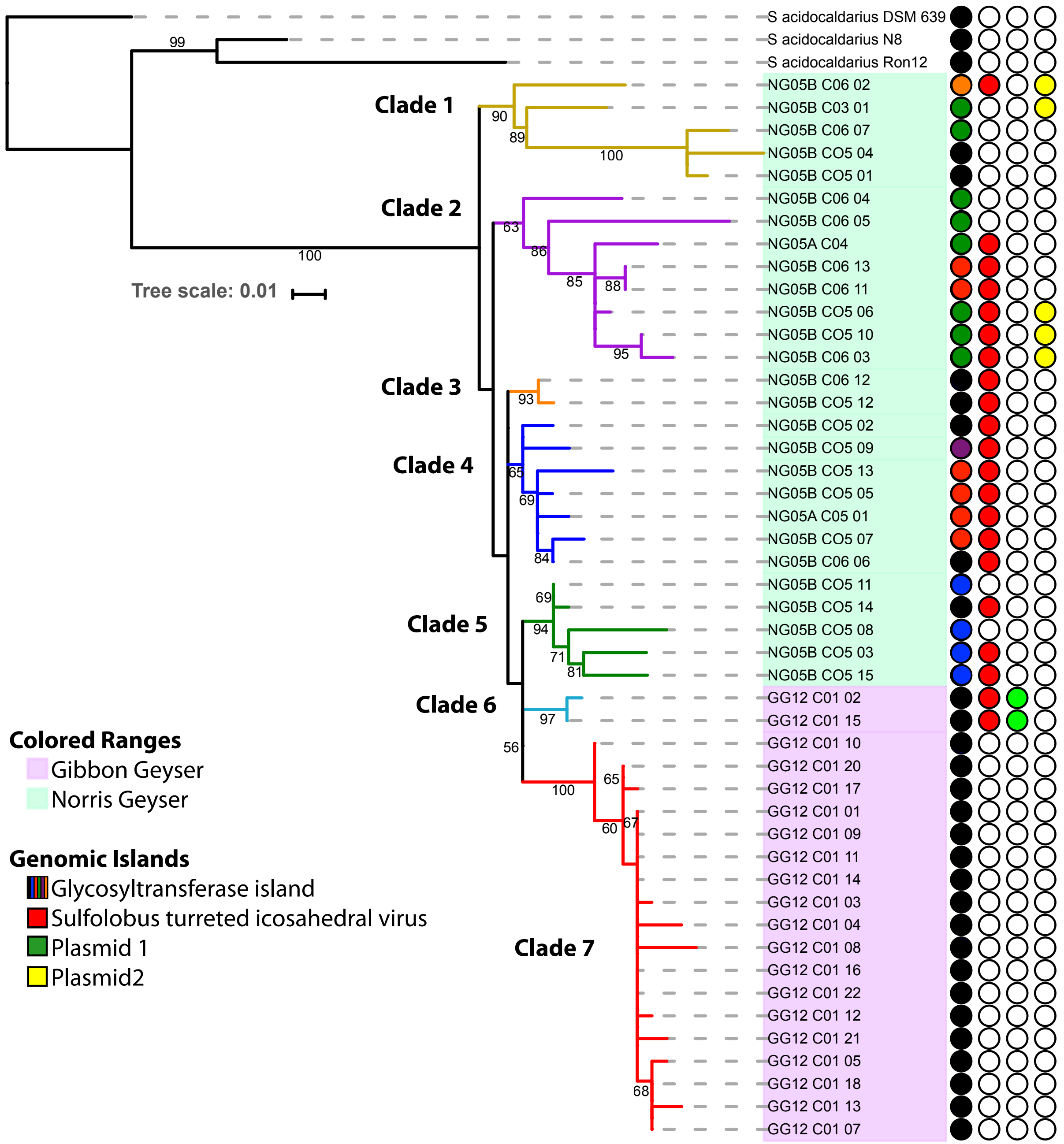

Supplementary Figure 3. Phylogenetic tree constructed by concatenating single nucleotide polymorphisms (SNPs) and insertions and deletions (indels) identified in the core genome relative to *S. acidocaldarius* DSM 639. The colored lines in the tree indicate each of the seven clades we identified among the S. acidocaldarius genomes isolated in 2012. Leaves are color-coded according to the hot spring from which they were isolated. Circles to the right of leaves indicate the presence or absence of specific genomic islands, and are colored as in Figure 1. Trees were visualized using the Interactive Tree of Life (ITOL) program (itol.embl.de).

Supplementary Table 4. Results from Tajima's Neutrality Test.

**Clade**	**M**	**S**	**p_s**	**Θ**	**Π**	**D**
1	5	31	0.260504	0.125042	0.121008	-0.241366
2	8	30	0.252101	0.097229	0.075030	-1.210254
4	7	18	0.151261	0.061739	0.045618	-1.458340
5	5	13	0.109244	0.052437	0.047059	-0.744854
7	19	19	0.159664	0.045682	0.021131	-2.058035

A total of 189 positions were included in the final dataset. Evolutionary analyses were conducted in MEGA5. Abbreviations: m = number of sequences, S = number of segregating sites, ps = S/m, theta = $ps/\sum_{i=1}^{n-1} 1/i$, pi = nucleotide diversity, and D is the Tajima test statistic. Clades with fewer than 3 genomes were not included in the analysis.

Supplementary Table 5. Single nucleotide polymorphisms (SNPs) that were fixed or polymorphic, synonymous or nonsynonymous within each clade.

**Clade 1**	**D**	**P**	**Genes with fixed nonsynonymous SNPs**
**N**	1	6	Saci_0886
**S**	0	25	
**Clade 2**	**D**	**P**	**Genes with fixed nonsynonymous SNPs**
**N**	0	13	
**S**	0	17	
**Clade 3**	**D**	**P**	**Genes with fixed nonsynonymous SNPs**
**N**	0	2	
**S**	0	3	
**Clade 4**	**D**	**P**	**Genes with fixed nonsynonymous SNPs**
**N**	0	9	
**S**	0	9	
**Clade 5**	**D**	**P**	**Genes with fixed nonsynonymous SNPs**
**N**	0	6	
**S**	2	7	
**Clade 6**	**D**	**P**	**Genes with fixed nonsynonymous SNPs**
**N**	3	1	Saci_0872, Saci_0886, Saci_2317
**S**	0	0	
**Clade 7**	**D**	**P**	**Genes with fixed nonsynonymous SNPs**
**N**	4	10	Saci_0406, Saci_0886, Saci_1274, Saci_1299, Saci_1780/1781
**S**	1	9	

Clades are labeled according to the clonal phylogeny shown in Figure 1. D: fixed in clade (present in all members of that clade and in no other strains); P: polymorphic in clade; N: nonsynonymous; S: synonymous. The fourth column lists the annotations relative to the reference strain (*S. acidocaldarius* DSM639) for the genes containing fixed nonsynonymous SNPs.

Coords in GG12_C01_01- start	Coords in GG12_C01_01- end	Matching gene in *S. acidocaldarius* DSM639	RAST Annotation	CDD annotation
1604767	1603454	Saci_1922	glycosyltransferase (ec 2.4.1.-)	Glycosyltransferase family 1 (cd/pfam/TIGR/COG)
1604997	1606148	Saci_1921	glycosyltransferase	Glycosyltransferase family 1 (cd/pfam/TIGR/COG)
1608357	1610798	Saci_1920	membrane protein	Predicted membrane protein (pfam/COG)
1613354	1610904	Saci_1919	membrane protein	Predicted membrane protein (pfam/COG)
1614554	1613895	Saci_1918	methyltransferase, fkbm family domain protein	methyltransferase (TIGR)
1614798	1617071	Saci_1917	hypothetical protein	No matches
1617103	1618425	Saci_1916	cell envelope, surface polysaccharides and lipopolysaccharides	Glycosyltransferase family 1 (cd/pfam/TIGR/COG)
1618440	1619396	Saci_1915	glycosyltransferase involved in cell wall biogenesis (ec 2.4.-.-)	Glycosyltransferase family 2 (cd/pfam/COG)
1620556	1619489	Saci_1914	glycosyltransferase (ec 2.4.1.-)	Glycosyltransferase family 1 (cd/pfam/TIGR/COG)
1620964	1621101	Saci_1913	hypothetical protein	No matches
1621176	1623506	Saci_1912	hypothetical protein	No matches
1624389	1623604	Saci_1911	hypothetical protein	Glycosyltransferase family 2 (cd)
1624499	1625491	Saci_1910	hypothetical protein	No matches
1626228	1625494	Saci_1909	dolichyl-phosphate mannose synthase related protein	Glycosyltransferase family 2 (cd/pfam/TIGR/COG)
1627852	1626326	Saci_1908	hypothetical protein	No matches
1629061	1628066	Saci_1907	glycosyltransferase (ec 2.4.1.-)	Glycosyltransferase family 1 (cd/pfam/TIGR/COG)
1630166	1632097	Saci_1906	membrane protein	No matches
1632767	1634152	Saci_1905	polysaccharide biosynthesis related protein	Wzx: exports metabolites across cell membranes/drug resistance (cd)
1634435	1635544	Saci_1904	glycosyltransferase, group 1 family protein	Glycosyltransferase family 1 (cd/pfam/TIGR/COG)
1636077	1637204	Saci_1903	conserved protein	No matches
1637455	1637847	Saci_1902	COG1720: Uncharacterized conserved protein	COG1720: Uncharacterized conserved protein [Function unknown]
1637903	1640950	Saci_1901	hypothetical proteins	No matches
1641112	1641759	Saci_1900	2-heptaprenyl-1,4-naphthoquinone methyltransferase (EC 2.1.1.163)	PRK05785: hypothetical protein; Provisional

Supplementary Table 6. Coordinates of ORFs identified in the glycosyltransferase-enriched genomic island in GG12_C01_01 using the RAST pipeline, as well as the corresponding gene in *S. acidocaldarius* DSM639 based on ORF clustering. Last two columns show annotations based on the RAST pipeline and RPSBLAST to the Conserved Domains Database.

Clade	Deletion Letter	Genome	Coords at which coverage drops below 10x	Coords at which coverage rises above 10x
1	D	NG05B_C03_01	1611124	1645967
1	A	NG05B_C05_04	full coverage	full coverage
1	F	NG05B_C06_02	1615794	1634891
1	D	NG05B_C06_07	1611126	1645972
1	A	NG05B_CO5_01	full coverage	full coverage
2	D	NG05A_C04	1611125	1645972
2	D	NG05B_C06_03	1611132	1645970
2	D	NG05B_C06_04	1611134	1645967
2	D	NG05B_C06_05	1611132	1645970
2	C	NG05B_C06_11	1614818	1637395
2	C	NG05B_C06_13	1614830	1637393
2	D	NG05B_CO5_06	1611125	1645970
2	D	NG05B_CO5_10	1611128	1645974
3	A	NG05B_C06_12	full coverage	full coverage
3	A	NG05B_CO5_12	full coverage	full coverage
4	C	NG05A_C05_01	1614819	1637393
4	A	NG05B_C06_06	full coverage	full coverage
4	A	NG05B_CO5_02	full coverage	full coverage
4	C	NG05B_CO5_05	1614828	1637393
4	C	NG05B_CO5_07	1614828	1637393
4	E	NG05B_CO5_09	1605295	1635634
4	C	NG05B_CO5_13	1614825	1637392
5	B	NG05B_CO5_03	1603770	1645021
5	B	NG05B_CO5_08	1603771	1645021
5	B	NG05B_CO5_11	1603771	1645022
5	A	NG05B_CO5_14	full coverage	full coverage
5	B	NG05B_CO5_15	1603771	1645021
6	A	GG12_C01_02	full coverage	full coverage
6	A	GG12_C01_15	full coverage	full coverage
7	A	GG12_C01_01	full coverage	full coverage
7	A	GG12_C01_03	full coverage	full coverage
7	A	GG12_C01_04	full coverage	full coverage
7	A	GG12_C01_05	full coverage	full coverage
7	A	GG12_C01_07	full coverage	full coverage
7	A	GG12_C01_08	full coverage	full coverage
7	A	GG12_C01_09	full coverage	full coverage
7	A	GG12_C01_10	full coverage	full coverage
7	A	GG12_C01_11	full coverage	full coverage
7	A	GG12_C01_12	full coverage	full coverage
7	A	GG12_C01_13	full coverage	full coverage
7	A	GG12_C01_14	full coverage	full coverage
7	A	GG12_C01_16	full coverage	full coverage
7	A	GG12_C01_17	full coverage	full coverage
7	A	GG12_C01_18	full coverage	full coverage
7	A	GG12_C01_20	full coverage	full coverage
7	A	GG12_C01_21	full coverage	full coverage
7	A	GG12_C01_22	full coverage	full coverage
ref	A	S_acidocaldarius_DSM639	full coverage	full coverage
ref	A	S_acidocaldarius_N8	full coverage	full coverage
ref	A	S_acidocaldarius_Ron12_I	full coverage	full coverage

Supplementary Table 7. Variation in the glycosyltransferase-enriched genomic island. Reads from each genome were mapped to a reference genomic island from GG12_C01_01. We identified six different patterns of variation in the genomic island; these are highlighted in different colors and given a letter designation in the second column. Fourth column shows coordinates relative to GG12_C01_01 for regions where coverage drops below 10x; fifth column shows coordinates where coverage rises above 10x. Coordinates and genes in the island are shown in Supplementary Table 6.

Annotation	ORF designation, STIV1	ORF designation, STIV2	Sequence length in *S. acidocaldarius* STIV (bp)	Sequence length in STIV1 (bp)	Sequence length in STIV2 (bp)	Coordinates in *S. acidocaldarius* STIV	Coordinates in STIV1	Coordinates in STIV2	% Identity with STIV1	% Identity with STIV2
Pyramid protein	C92	Not present	279	279	Not present	2,931-3,209	3,888-4,166	Not present	88.5	Not present
Integrase	A510	A510	1533	1533	1,530	3,403-4935	2,155-3,684	2,167-3,699	74.9	74.4
Major coat protein	B345	A345	1,038	1,038	1,038	8,839-9,876	14,942-15,979	14,038-15,075	81.6	72.7
Transcription factor	B116	B116	354	351	351	15,711-16,064	7,994-8,344	8,165-8,515	71.7	60.2
Turret protein	A223	B631	678	672	1,895	1,153-1,830	5,260-5,931	5,291-7,186	82.3	66bp in N-terminus match with 89.4%
Glycosyltransferase	A197	B197	594	594	593	1,869-2,462	4,624-5,217	4652-5245	80.9	74.5
Turret protein	C557	C510	1,644	1,674	1,533	7,184-8,227	15,990-17,663	15,090-16,622	36.0	49.8
Hypothetical protein	C121	C96	270	366	290	12,914-12,645	11,616-11,981	11,139-11,429	79.5	95.5 % at the N-terminus and 78.6 % at the C-terminus
Turret protein	C381	Not present	1,155	1,146	Not present	1-1,155	5,931-7,076	Not present	79.1	Not present

ATPase	B164	B204	615	495	614	10,274-10,888	13,958-14,452	13,157-13,771	78.1	77.6
Virion protein	B130	C141	426	393	425	5,990-6,415	797-1,189	792-1217	71.9	71.8
Virion protein	A55	A55	168	168	167	5,808-5,975	1,201-1,368	1,222-1,389	80.0	59.0

Supplementary Table 8. Summary of predicted open reading frames in the Sulfolobus turreted icosahedral virus (STIV) integrated in *S. acidocaldarius*, and comparisons with open reading frames in previously sequenced STIV genomes.

Genome Name	NCBI Accession	Coords1	Coords2	Comments	CRISPR locus
S. islandicus M.14.25	NC_012588	550085	550061	dTDP-4-dehydrorhamnose reductase	CRISPR 3 spacer 27
S. islandicus M.14.25	NC_012588	912187	912224	hypothetical protein	CRISPR 3 spacer 75
S. islandicus L.S.2.15	NC_012589	1576643	1576609	hypothetical protein	CRISPR 3 spacer 75
S. islandicus L.S.2.15	NC_012589	1576660	1576696	hypothetical protein	CRISPR 4 spacer 5
S. islandicus Y.G.57.14	NC_012622	1504835	1504857	hypothetical protein, close to plasmid genes	CRISPR 4 spacer 7
S. islandicus Y.G.57.14	NC_012622	1484143	1484170	close to TraD and other plasmid genes	CRISPR 4 spacer 12
S. islandicus Y.G.57.14	NC_012622	1485554	1485587	close to TraD and other plasmid genes	CRISPR 4 spacer 77
S. islandicus Y.N.15.51	NC_012623	1843072	1843045	ABC transporter	CRISPR 3 spacer 25
S. islandicus Y.N.15.51	NC_012623	788977	788955	hypothetical protein, close to plasmid genes	CRISPR 4 spacer 7
S. islandicus Y.N.15.51 plasmid	NC_012624	34222	34205	Located on a plasmid chromosome	CRISPR 4 spacer 7
S. islandicus Y.N.15.51 plasmid	NC_012624	12410	12390	Located on a plasmid chromosome	CRISPR 4 spacer 7
S. islandicus Y.N.15.51 plasmid	NC_012624	40190	40208	Located on a plasmid chromosome	CRISPR 4 spacer 7
S. islandicus Y.N.15.51 plasmid	NC_012624	24657	24690	Located on a plasmid chromosome	CRISPR 4 spacer 77
S. islandicus L.D.8.5 plasmid	NC_013770	10444	10427	Located on a plasmid chromosome	CRISPR 4 spacer 7
S. islandicus L.D.8.5 plasmid	NC_013770	12929	12912	Located on a plasmid chromosome	CRISPR 4 spacer 7
S. islandicus L.D.8.5 plasmid	NC_013770	20497	20479	Located on a plasmid chromosome	CRISPR 4 spacer 7
S. islandicus L.D.8.5 plasmid	NC_013770	946	978	Located on a plasmid chromosome	CRISPR 4 spacer 77
S. islandicus L.D.8.5 plasmid	NC_013770	20084	20060	Located on a plasmid chromosome	CRISPR 4 spacer 81
S. islandicus M.16.27	NC_012632	573664	573640	dTDP-4-dehydrorhamnose reductase	CRISPR 3 spacer 27
S. islandicus M.16.27	NC_012632	1479971	1480008	hypothetical protein, close to plasmid genes	CRISPR 3 spacer 75
S. islandicus M.16.27	NC_012632	1463479	1463447	hypothetical protein, close to plasmid genes	CRISPR 3 spacer 76
S. islandicus REY15A	NC_012726	572924	572900	dTDP-4-dehydrorhamnose reductase [Cell envelope	CRISPR 3 spacer 27

				biogenesis, outer membrane]; Region: RfbD; COG1091	
S. islandicus L.D.8.5	NC_013769	1369968	1369933	ATPase AAA	CRISPR 3 spacer 8
S. islandicus L.D.8.5	NC_013769	1591678	1591644	hypothetical protein	CRISPR 3 spacer 75
S. islandicus L.D.8.5	NC_013769	1591695	1591731	hypothetical protein	CRISPR 4 spacer 5
S. islandicus L.D.8.5	NC_013769	1376911	1376933	hypothetical protein, close to plasmid genes	CRISPR 4 spacer 7
S. islandicus L.D.8.5	NC_013769	1343520	1343541	hypothetical protein, close to plasmid genes	CRISPR 4 spacer 7
S. islandicus L.D.8.5	NC_013769	1872713	1872692	hypothetical protein, close to plasmid genes	CRISPR 4 spacer 7
S. islandicus L.D.8.5	NC_013769	1340990	1341022	hypothetical protein, close to plasmid genes	CRISPR 4 spacer 77
S. islandicus L.D.8.5	NC_013769	1880391	1880367	hypothetical protein, close to plasmid genes	CRISPR 4 spacer 81
S. islandicus HVE10.4	NC_017275	402805	402781	dTDP-6-deoxy-L-lyxo-4-hexulose reductase and	CRISPR 3 spacer 27
S. islandicus HVE10.4	NC_017275	1798731	1798768	hypothetical protein, close to many IS elements	CRISPR 3 spacer 75
S. islandicus HVE10.4	NC_017275	724373	724354	intergenic, near toxin/antitoxin region	CRISPR 4 spacer 5
S. islandicus HVE10.4	NC_017275	1531070	1531034	hypothetical protein, close to plasmid genes	CRISPR 4 spacer 5
S. islandicusREY15A	NC_017276	772183	772146	hypothetical protein - close to many IS elements	CRISPR 3 spacer 75
S. islandicusREY15A	NC_017276	1662559	1662596	intergenic region	CRISPR 3 spacer 75
S. islandicusREY15A	NC_017276	2486773	2486795	transposase/IS element	CRISPR 4 spacer 94
S. islandicus rod-shaped virus 2	NC_004086	21734	21770	Located on a viral genome	CRISPR 4 spacer 117
S. islandicus rod-shaped virus 1	NC_004087	18268	18302	Located on a viral genome	CRISPR 4 spacer 117

Supplementary Table 9. Matches between CRISPR spacers in S. acidocaldarius and S. islandicus genomes and Sulfolobus viral genomes. All matches had an evalue of 0.001 or lower.
